# Comparison of anthracycline-containing and anthracycline-free regimens in neoadjuvant HER-2 positive breast cancer treatment

**DOI:** 10.1038/s41598-024-61562-w

**Published:** 2024-05-09

**Authors:** Murat Bardakci, Hilal Karakas, Dogan Bayram, Nilufer Avci, Sait Kitapli, Mirac Ozen, Ferit Aslan, Caglar Koseoglu, Ahmet Kadioglu, Ilknur D. Onur, Teoman Sakalar, Mahmut Buyuksimsek, Ali Alkan, Yakup Ergun, Ali O. Kaya, Burak Bilgin, Bulent Yalcin

**Affiliations:** 1 Department of Medical Oncology, Ankara City Hospital, 1604. Street, No: 9, 06000 Ankara, Turkey; 2 Department of Medical Oncology, Medicana Bursa Hospital, Bursa, Turkey; 3 Department of Medical Oncology, Faculty of Medicine, Mugla Sitki Kocman University, Mugla, Turkey; 4 Department of Medical Oncology, Faculty of Medicine, Sakarya University, Sakarya, Turkey; 5 Department of Medical Oncology, Medicalpark Ankara Batikent Hospital, Ankara, Turkey; 6 Department of Medical Oncology, Ankara Gülhane Training and Research Hospital, Ankara, Turkey; 7 Department of Medical Oncology, Abdurrahman Yurtaslan Ankara Oncology Training and Research Hospital, Ankara, Turkey; 8 Department of Medical Oncology, Kahramanmaras Necip Fazil City Hospital, Kahramanmaras, Turkey; 9 Department of Medical Oncology, Adana City Training and Research Hospital, Adana, Turkey; 10 Department of Medical Oncology, Antalya City Hospital, Antalya, Turkey; 11 Department of Medical Oncology, Medicana International Beylikduzu Hospital, Istanbul, Turkey

**Keywords:** Cancer, Medical research, Oncology

## Abstract

While some clinics have adopted abbreviated neoadjuvant treatment for HER2-positive breast cancer, there remains a shortage of comprehensive clinical data to support this practice. This is a retrospective, multicenter study. A total of 142 patients were included in the study who are HER2-positive breast cancer, aged ≤ 65 years, with left ventricular ejection fraction ≥ 50%, received neoadjuvant chemotherapy and underwent surgery at 10 different oncology centers in Türkiye between October 2016 and December 2022. The treatment arms were divided into 4–6 cycles of docetaxel/trastuzumab/pertuzumab for arm A, 4 cycles of adriamycin/cyclophosphamide followed by 4 cycles of taxane/TP for arm B. There were 50 patients (35.2%) in arm A and 92 patients (64.8%) in arm B. The median follow-up of all of the patients was 19.9 months (95% CI 17.5–22.3). The 3-year DFS rates for treatment arms A and B were 90.0% and 83.8%, respectively, and the survival outcomes between the groups were similar (*p* = 0.34). Furthermore, the pathologic complete response rates were similar in both treatment arms, at 50.0% and 51.1%, respectively (*p* = 0.90). This study supports shortened neoadjuvant treatment of HER2-positive breast cancer, a common practice in some clinics.

## Introduction

The human epidermal growth factor receptor 2 (HER2) protein is often overexpressed in 18–20% of breast cancer cases due to the amplification of the gene responsible for HER2, located on chromosome 17^1^. This particular subtype of breast cancer is known for its aggressive clinical behavior, characterized by high recurrence and metastasis rates, a poor prognosis, and a 5-year survival rate of less than 30%^2^. Fortunately, anti-HER2 targeted therapy has significantly improved the prognosis for patients with HER2-positive breast cancer, particularly when combining treatments like trastuzumab and pertuzumab^3^.

International research efforts are currently focused on a shortened neoadjuvant regimen followed by tapered adjuvant therapy for HER2-positive breast cancer. However, there is a lack of sufficient studies to determine the feasibility of this approach. The clinical benefit of combining the HER2-targeted monoclonal antibodies pertuzumab and trastuzumab was initially demonstrated in patients with HER2-positive metastatic breast cancer who had experienced disease progression during previous trastuzumab treatment^4^. Building on these results, pertuzumab, trastuzumab, and chemotherapy were tested in the HER2-positive neoadjuvant setting in trials like NeoSphere and TRYPHAENA. In the NeoSphere study, patients with operable, locally advanced, or inflammatory HER2-positive breast cancer received neoadjuvant docetaxel/trastuzumab/pertuzumab (DTP) therapy, with the primary endpoint being the achievement of a pathologic complete response (pCR). DTP therapy led to higher pCR rates (45.8%)^5^. The TRYPHAENA study, a randomized, multicenter trial, primarily aimed to evaluate the tolerability and cardiac safety of dual blockade neoadjuvant therapy. The pCR rates were similar between the groups, and the anthracycline-free group exhibited slightly lower cardiotoxicity rates^6^. It is worth noting that HER signaling plays a role in myocardial homeostasis, and trastuzumab treatment has been associated with cardiac dysfunction, particularly when combined with higher cumulative doses of anthracyclines^7^. The neoadjuvant setting is ideal for evaluating the safety and activity of drugs as it offers early indications of their performance over a relatively short period, with pCR serving as a proxy for long-term treatment outcomes^8^. In another study involving patients with HER2-positive breast cancer, a neoadjuvant regimen of weekly paclitaxel for 12 weeks and 4 cycles of trastuzumab/pertuzumab (TP) every 3 weeks was administered. Patients who achieved pCR subsequently received only adjuvant TP, demonstrating the feasibility of transitioning from multi-agent cytotoxic chemotherapy to single-agent cytotoxic chemotherapy when combined with dual anti-HER2 antibody therapy in patients with pCR after neoadjuvant paclitaxel/TP^9^. In cases where patients are of advanced age or have cardiac issues, anthracycline-free treatments are often preferred.

This study aimed to compare pCR rates and survival outcomes between reduced adjuvant antibody dual therapy (TP, without chemotherapy) and standard therapy in patients aged 65 or younger, with a left ventricular ejection fraction (LVEF) of 50% or higher, who underwent surgery following neoadjuvant DTP treatment.

## Methods

### Patients

A retrospective analysis was conducted of data from breast cancer patients who were HER2 positive [immunohistochemistry (IHC) 3+ or IHC (2+) and fluorescence in situ hybridization (FISH+)] and had undergone neoadjuvant chemotherapy followed by surgery at 10 different oncology centers in Türkiye, between October 2016 and December 2022. The study aimed to investigate specific patient profiles and treatment outcomes. The following inclusion criteria were applied: (1) age at diagnosis 18–65 years; (2) LVEF ≥ 50%; (3) no previous history of malignancy; (4) Eastern Cooperative Oncology Group (ECOG) performance status of 0–1; (5) stage (T_1–4_N_0–3_M_0_) patients; (6) having received 4–6 cycles of neoadjuvant DTP or 4 cycles of adriamycin/cyclophosphamide (AC) followed by 4 cycles of taxan/TP chemotherapy; (7) having been operated on for HER2-positive breast cancer. The staging was determined according to the American Joint Committee on Cancer primary Tumor, regional lymph Nodes, and distant Metastasis (TNM) Staging Classification for Breast Cancer. A total of 152 patients meeting these inclusion criteria were included in the study. Additionally, the presence of comorbidities such as diabetes mellitus, hypertension, ischemic heart disease, hypothyroidism, sarcoidosis, rheumatoid arthritis, psoriasis, hepatitis B infection, and familial Mediterranean fever was noted. The patients’ physical conditions were assessed, including their ECOG performance status and the documentation of adverse events using the National Cancer Institute Common Terminology Criteria (NCI-CTC) Adverse Events (version 5.0). Follow-up assessments were conducted post-treatment every 3 months for the first 2 years, every 6 months from years 2–5, and annually thereafter.

Patients were categorized as ECOG (0/1), T stage (T_1–2_/T_3–4_), N stage (N_0–1_/N_2–3_), clinical stage [operable (T_0–3_N_1_M0 or T_1–3_N_0–1_M_0_), locally advanced (T_0–4_N_2–3_M_0_), inflammatory (T_4d_N_0–3_M_0_)], hormone receptor status (ER and/or PR positive-ER and PR negative), and grade (GX-2/G3). Sociodemographic and tumor characteristics, treatment adverse effects, and treatment outcomes were recorded for all of the patients based on the treatment modality they received. The main focus of the study was on evaluating the pCR, DFS, and adverse effects to draw comparisons and gain insight.

### Treatment modalities

Arm A: DTP; docetaxel 75–100 mg/m^2^ IV, day 1; trastuzumab treatment loading 8 mg/kg, maintenance 6 mg/kg, pertuzumab treatment loading 840 mg, maintenance 420 mg; day 1, cycle length: 21 days. Arm B: AC; doxorubicin 60 mg/m^2^ IV, cyclophosphamide 600 mg/m^2^, day 1, cycle length: 21 days, taxan (docetaxel 75–100 mg/m^2^ IV or paclitaxel 80 mg mg/m^2^/week); trastuzumab treatment loading 8 mg/kg, maintenance 6 mg/kg, pertuzumab treatment loading 840 mg, maintenance 420 mg; day 1, cycle length: 21 days.

In both treatment arms, adjuvant trastuzumab emtansine (T-DM1) treatment was given for 1 year to patients with postoperative residual disease if approval could be obtained, taking into account Türkiye’s reimbursement conditions. When approval could not be obtained, trastuzumab +/− pertuzumab treatment was given. Patients with pCRs received trastuzumab +/− pertuzumab treatment for a total of 1 year. After surgical and axillary staging in both treatment arms, the patients were consulted by radiation oncology for the necessity of radiotherapy treatment. Adjuvant endocrine therapy was added for the hormone-positive patients and adjuvant radiotherapy was given to appropriate patients. In arm B, adjuvant treatment could not be completed in 1 patient due to optic neuritis after trastuzumab treatment and in 1 patient due to treatment refusal.

### Statistical analysis

Statistical analysis was performed using IBM SPSS Statistics for Windows 25.0 (IBM Corp., Armonk, NY, USA. Two groups were compared with the Mann–Whitney U test and Pearson chi-squared or Fisher test for continuous and categorical variables, respectively. Survival analysis was analyzed by the Kaplan–Meier method using the long-rank test. Survival times were determined within the 95% confidence interval (CI) range. *p* < 0.05 was considered statistically significant. DFS was defined as the time from the date of operation to the date of relapse or death from any cause, and as the time to the last follow-up date for the survivors.

### Statement of ethics

This study was approved by the institutional ethics committee. All procedures followed were by the ethical standards of the responsible committee and the latest Declaration of Helsinki. Informed consent was not obtained from the patients due to the retrospective study design. The Ankara City Hospital Ethics Committee approved the non-requirement of informed consent. This study protocol was reviewed and approved by Ankara City Hospital Ethics Committee No.1, approval number: E1-23-4214/Date: 01.11.2023.

## Results

### Patient characteristics

A total of 142 patients were included in the study. There were 50 patients (35.2%) in arm A and 92 patients (64.8%) in arm B. The mean age of the patients in arm A was 53 years (± 11, SD), while in arm B it was 48 years (± 11, SD). The baseline patient characteristics are presented in Table 1. There were no significant differences in the clinical and pathological characteristics between the treatment arms. No significant difference was observed between the treatment arms in terms of T-stage or N-stage, which could potentially affect the outcomes. During the treatment period, an LVEF decline was observed in 3 patients (6%) in arm A and in 11 patients (12%) in arm B. Although the LVEF decreases were asymptomatic in both treatment arms, the rate of LVEF decrease was between 11 and 20% in 2 patients (2.2%) in arm B. Various grades of thrombocytopenia were seen in 54.0% of patients in arm A and 34.0% in arm B. However, thrombocytopenia at grade 3 or higher was only seen in arm A (*p* = 0.04). Adverse events according to the treatment arm are presented in Tables 2 and 3.

**Table 1:** Patient and tumor characteristics according to the treatment group at the time of diagnosis.

	Arm A	Arm B	*p* value
	n (%)	n (%)	
Age (mean, SD)	53 (± 11)	48 (± 11)	0.01^b^
Comorbidity	20 (40.0)	32 (34.8)	0.53
ECOG			0.06
0	34 (68.0)	48 (52.2)	
1	16 (32.0)	44 (47.8)	
Clinical T stage			0.58
T1–2	38 (76.0)	66 (71.7)	
T3–4	12 (24.0)	26 (28.3)	
Clinical N stage			0.58
N0	5 (10.0)	11 (12.0)	
N1	15 (30.0)	37 (40.2)	
N2	23 (46.0)	34 (37.0)	
N3	7 (14.0)	10 (10.8)	
Clinical stage			0.14
Operable	20 (40.0)	45 (48.9)	
Locally advanced	30 (60.0)	43 (46.7)	
Inflammatory	0 (0.0)	4 (4.3)	
Hormone status			0.99
ER and/or PR-positive	31 (62.0)	57 (62.0)	
ER and PR-negative	19 (38.0)	35 (38.0)	
Grade			0.47
Gx/G1/G2	26 (52.0)	42 (45.7)	
G3	24 (48.0)	50 (54.3)	
Ki-67 level^a^ (%)	30 (7–90)	40 (8–95)	0.66
Lymphovascular invasion	21 (42.0)	41 (44.6)	0.76
Perineural invasion	15 (30.0)	22 (23.9)	0.43
Number of cycles^a^	4 (3–6)	8 (6–8)	< 0.001^b^
Neoadjuvant treatment completion	46 (92.0)	88 (95.7)	0.45

^a^Presented with the median instead of n, min–max instead of %.

^b^Statistically significant.

**Table 2:** Adverse effects according to the treatment group (any grade).

	Arm A	Arm B	*p* value
	n (%)	N (%)	
Decreased EF during treatment	3 (6.0)	11 (12.0)	0.37
EF decrease rate			0.50
0–10%	3 (6.0)	9 (9.8)	
11–20%	0 (0.0)	2 (2.2)	
EF decrease (asymptomatic)	3 (6.0)	11 (12.0)	0.37
Anemia	33 (66.0)	54 (58.7)	0.39
Thrombocytopenia	27 (54.0)	28 (30.4)	0.006^a^
Lokopenia	35 (70.0)	51 (55.4)	0.09
Neutropenia	35 (70.0)	60 (65.2)	0.56
Nausea	39 (78.0)	76 (82.6)	0.50
Vomiting	27 (54.0)	58 (63.0)	0.29
Mucositis	26 (52.0)	32 (34.8)	0.04^a^
Fatigue	46 (92.0)	81 (88.0)	0.46
Diarrhea	18 (36.0)	32 (34.8)	0.88
Rash	10 (20.0)	18 (19.6)	0.95
Allergy and anaphylaxis	5 (10.0)	7 (7.6)	0.75
Hepatotoxicity	6 (12.0)	16 (17.4)	0.39
Neuropathy	24 (48.0)	46 (50.0)	0.82
Febrile neutropenia	2 (4.0)	1 (1.1)	0.28
Toxicity-induced dose reduction	9 (18.0)	11 (12.0)	0.32
Hospitalization due to toxicity	8 (16.0)	15 (16.3)	0.96

^a^Statistically significant.

**Table 3:** Grade-3 and higher adverse event results according to the treatment group.

	Arm A	Arm B	*p* value
	n (%)	n (%)	
Anemia	3 (6.0)	6 (6.5)	0.99
Thrombocytopenia	3 (6.0)	0 (0.0)	0.04^a^
Lokopenia	1 (2.0)	4 (4.3)	0.65
Neutropenia	3 (6.0)	9 (9.8)	0.54
Nausea	6 (12.0)	10 (10.9)	0.83
Vomiting	2 (4.0)	5 (5.4)	0.99
Mucositis	1 (2.0)	0 (0.0)	0.35
Fatigue	9 (18.0)	10 (10.9)	0.23
Diarrhea	3 (6.0)	8 (8.7)	0.74
Rash	1 (2.0)	1 (1.1)	0.99
Hepatotoxicity	1 (2.0)	0 (0.0)	0.35
Neuropathy	1 (2.0)	1 (1.1)	0.99

^a^Statistically significant.

The breast conserving surgery (BCS) rates were 44% and 44.6%, axillary lymph node dissection (ALND) rates were 34% and 34.8%, and the groups were similar in terms of the type of operation and axillary staging (*p* = 0.92) for treatment arms A and B, respectively. During the follow-up period, 1 patient in arm A (local or distant metastasis) and 5 patients in arm B (local or distant metastasis) developed recurrence. The patients in arm A were hormone-positive/HER2-negative, while the patients in arm B were hormone-negative/HER2-positive. Surgical outcomes according to treatment group are presented in Table 4.

**Table 4:** Surgical outcomes, neoadjuvant treatment responses, and adjuvant treatment types according to the treatment group.

	Arm A	Arm B	*p* value
	n (%)	n (%)	
Operation type			0.94
BCS	22 (44.0)	41 (44.6)	
MRM	28 (56.0)	51 (55.4)	
Axilla staging			0.92
SLND	33 (66.0)	60 (65.2)	
ALND	17 (34.0)	32 (34.8)	
Complete response (ypT0N0)	25 (50.0)	47 (51.1)	0.90
Surgical margin positivity	0 (0.0)	1 (1.2)	0.70
Grade			0.12
Gx	18 (36.0)	45 (48.9)	
G1	11 (22.0)	9 (9.8)	
G2	17 (34.0)	26 (28.3)	
G3	4 (8.0)	12 (13.0)	
Lymphovascular invasion	9 (23.7)	22 (25.6)	0.82
Perineural invasion	3 (7.9)	13 (15.1)	0.38
Adjuvant treatment			0.001^a^
AC + trastuzumab	2 (4.0)	0 (0.0)	
Trastuzumab	34 (68.0)	75 (81.5)	
Trastuzumab + pertuzumab	12 (24.0)	5 (5.4)	
Trastuzumab emtansine (T-DM1)	2 (4.0)	12 (13.0)	
Adjuvant treatment completion			0.08
Yes	42 (84.0)	85 (92.4)	
No	0 (0.0)	2 (2.2)	
Continues	8 (16.0)	5 (5.4)	
Relapse status	1 (2.0)	5 (5.4)	0.42
Relapse site			0.66
Local	0 (0.0)	1 (1.1)	
Liver	0 (0.0)	1 (1.1)	
Lymph node	0 (0.0)	2 (2.1)	
Central nervous system	0 (0.0)	1 (1.1)	
Local-bone-lymph node	1 (2.0)	0 (0.0)	
Relapse ER/PR status			0.16
ER and/or PR-positive	1 (2.0)	0 (0.0)	
ER and PR-negative	0 (0.0)	5 (5.4)	
Relapse HER-2 status			0.50
Positive	0 (0.0)	4 (4.3)	
Negative	1 (2.0)	1 (1.1)	

*BCS* breast conserving surgery, *MRM* modified radical mastectomy, *SLND* sentinel lymph node dissection, *ALND* axillary lymph node dissection, *AC* doxorubicin and cyclophosphamide.

^a^Statistically significant.

### Survival analysis and treatment effect

**Figure 1:**
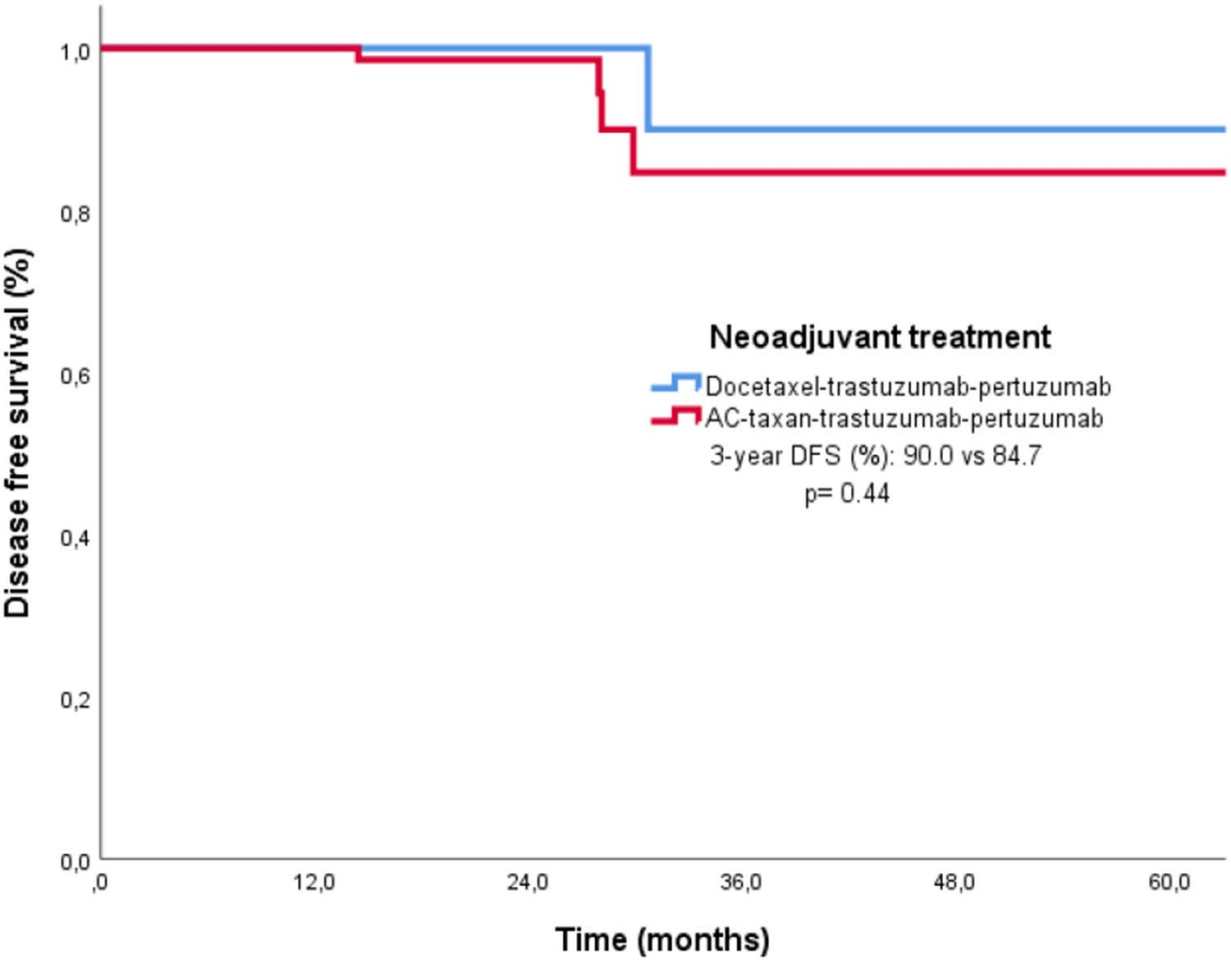
Kaplan Meier analysis of DFS by treatment arm.

The median follow-up of all of the patients was 19.9 months (95% CI 17.5–22.3). The median DFS was not reached in either treatment arm. The 3-year DFS rates for treatment arms A and B were 90.0% and 83.8%, respectively, and the survival outcomes between the groups were similar (*p* = 0.34). Kaplan–Meier survival analysis according to treatment arm is shown in Fig. 1. In addition, the pCR rates were similar in both treatment arms, at 50.0% and 51.1%, respectively (*p* = 0.90). In arm A, 26 patients (52.0%) received 4 cycles of DTP treatment, while 24 patients (48.0%) received 6 cycles, and there was no difference in DFS between the treatment arms (*p* = 0.48). The pCR status of the patients according to the treatment arms and the adjuvant treatments received by the patients are shown in Table 5.

**Table 5:** The pCR status of patients according to treatment arms and adjuvant treatments received by the patients.

	Arm-A		*p* value	Arm-B		*p* value
	pCR	Non-pCR		pCR	Non-pCR	
Adjuvant treatment	n (%)	n (%)	0.32	n (%)	n (%)	< 0.001
AC + Trastuzumab	0 (0.0)	2 (8.0)		0 (0.0)	0 (0.0)	
Trastuzumab	18 (72.0)	16 (64.0)		44 (93.6)	31 (68.9)	
Trastuzumab + Pertuzumab	7 (28.0)	5 (20.0)		3 (6.4)	2 (4.4)	
Trastuzumab emtansin (T-DM1)	0 (0.0)	2 (8.0)		0 (0.0)	12 (26.7)	

*AC* doxorubicin and cyclophosphamide, *pCR* pathological complete response.

## Discussion

This study has demonstrated the feasibility of using the DTP regimen for the treatment of HER2-positive breast cancer, showing comparable pCR rates and DFS rates between neoadjuvant DTP and the standard AC-taxan/TP treatments. In a relatively small cohort of patients, 1 patient experienced a relapse with DTP treatment, while 5 patients experienced relapses with standard treatment during a short follow-up period. These findings suggest that the DTP regimen may offer advantages by avoiding toxicities associated with standard combination chemotherapy regimens.

The pCR rates of 50.0% observed with neoadjuvant DTP in this study are in line with other studies that have explored HER2-positive breast cancer treatments, including various chemotherapy plus TP regimens. For instance, the KRISTINE study^10^ reported pCR (ypT_0_N_0_) rates of 55.7% and 44.4% when patients received 6 cycles of docetaxel/carboplatin/TP or T-DM1/P, and the TRYPHAENA study^6^ reported pCR rates of 45.3% and 51.9% when patients received 6 cycles of 5-fluorouracil/epirubicin/cyclophosphamide-DTP or docetaxel/carboplatin/TP, respectively. Meta-analyses have shown a relationship between pCR and event-free survival^11^. In the NeoSphere study^5^, patients with operable, locally advanced, or inflammatory HER2-positive breast cancer were randomized into 4 arms (arm A: trastuzumab and docetaxel, arm B: pertuzumab, trastuzumab, and docetaxel, arm C: pertuzumab and trastuzumab, and arm D: pertuzumab and docetaxel). After the operations, the patients received adjuvant chemotherapy and trastuzumab (for 1 year). For 12 weeks of neoadjuvant treatment, combining pertuzumab with trastuzumab plus docetaxel achieved a pCR rate of 45.8%. The NeoALTTO^12^, CHER-LOB^13^, and NSABP B-41^14^ studies combined trastuzumab with lapatinib for 18, 26, and 28 weeks of neoadjuvant treatment, respectively, resulting in pCR rates of 51.3%, 46.7%, and 62.0%. In the current study, a pCR rate of 51.1% was achieved with AC plus DTP, which aligns with the outcomes of other studies. HER2-positive breast cancer is well suited to the reduction of systemic therapy due to the development of highly effective targeted therapies with relatively low toxicity. Although pCR is used as the last surrogate for systemic therapy reduction, the concern about the toxicity of combination chemotherapy in patients without pCR and the fact that the most important reason cited for reducing this concern is the planned use of T-DM1 reflects the fact that it is easier to consider toxic therapy reduction by substituting a more targeted, less toxic agent for the standard combination chemotherapy regimen. Cancer therapy-associated cardiac dysfunction has been described in 3–10% of early-stage breast cancer patients treated with trastuzumab and in 19% treated with trastuzumab plus anthracyclines^15^. For dual HER2 blockade, cardiotoxicity may be synergistic. Specifically, dual HER2 blockade combining trastuzumab and pertuzumab resulted in a higher incidence of heart failure compared to trastuzumab alone^16^. In the present study, the LVEF reduction in both treatment arms was 6.0% and 12%, respectively, and numerically less in the DTP arm. Although not statistically significant, LVEF decreased between 11 and 20% in two patients in the standard treatment arm. In the APHINITY study, the 3-year DFS was 92.0% in node-positive early stage HER2-positive breast cancer patients treated with TP plus adjuvant chemotherapy^17^. The TRYPHAENA study was a randomized, multicenter trial whose primary objective was to evaluate the tolerability, especially cardiac safety, of dual-blockade neoadjuvant therapy. In that study, the patients were randomized into 3 arms (arm A: anthracycline-based chemotherapy and dual-anti-HER2 therapy followed by docetaxel and dual anti-HER-2 therapy, arm B: anthracycline-based therapy followed by docetaxel and dual anti-HER-2 therapy, and arm C: chemotherapy without anthracycline (taxan + platinum) and dual anti-HER-2 therapy). The pCR rates were similar between the groups and cardiotoxicity rates were slightly lower in the anthracycline-free group. The 3-year DFS with neoadjuvant docetaxel/carboplatin/TP treatment was 90.0%^18^. In the current study, the 3-year DFS with DTP treatment was 90.0% compared to 83.8% with standard treatment. It is believed that these comparable survival results may save patients from additional chemotherapy toxicity. In another study with 98 patients, treatment naive, stage II–III, HER2-positive breast cancer patients received neoadjuvant weekly paclitaxel (12 weeks) and 4 cycles of TP every 3 weeks. The primary endpoint of the study was the receipt of adjuvant cytotoxic chemotherapy not directed against HER2. Patients who failed to achieve pCR radiologically and clinically received preoperative doxorubicin and cyclophosphamide, with an overall pCR rate of 56.7%. In this study, adjuvant TP was given to patients who achieved pCR. This study demonstrated the feasibility of regression from multi-agent cytotoxic chemotherapy to single agent cytotoxic chemotherapy in combination with dual anti-HER2 antibody therapy in patients with pCR after neoadjuvant paclitaxel/TP^9^.

This study had several important limitations that should be considered. First, it was a retrospective study, which means that it relied on historical data and patient records. Additionally, it included a relatively small number of patients, which can limit the generalizability of the findings. An additional limitation of our study is that the follow-up period was relatively short, so the number of events to determine survival outcomes was low. Longer follow-up periods are required for DFS and OS data to mature. Another notable limitation was the lack of standardized use of anti-HER2 therapies due to reimbursement conditions in Türkiye. This variable usage of anti-HER2 therapies could have introduced potential biases in the study’s results. Despite these limitations, the study’s findings are still valuable, as they provide support for the concept of reduced neoadjuvant cytotoxic treatment, a practice that has already been implemented in some clinics. These results contribute to the ongoing discussion around more personalized and less toxic treatment approaches for HER2-positive breast cancer, even within the constraints of the study’s limitations.

This study provides support for the adoption of a shortened neoadjuvant treatment approach for HER2-positive breast cancer, which is already a common practice in some clinical settings. The findings, showing comparable DFS and pCR rates between the DTP regimen and the standard AC-taxan/TP treatments, suggest that DTP may offer the advantage of reducing the severe toxicities associated with the traditional combined chemotherapy regimens. This study’s results contribute to the growing body of evidence supporting the use of more targeted and less toxic approaches in the treatment of HER2-positive breast cancer, ultimately improving the quality of care and patient outcomes.

## Data Availability

All data generated or analyzed during this study were included in this article. Further inquiries can be directed to the corresponding author.
